# A Stable Vitamine

**Published:** 1920-04-24

**Authors:** 


### A Stable Vitamine.

In the February number of the American *Journal of Medical Sciences* an interesting account is given by Dubin and Lewi of a method by which may be prepared a stable vitamine product which can prove effective therapeutically in increasing bodily growth and vigour when the nutritive powers of the individual are below normal. If it be assumed that malnutrition depends not only upon an insufficiency of food but upon an improper combination of foods or improperly prepared foods—facts which have been conclusively demonstrated of late—then the value in a preparation of a stable compound containing the three essential vitamines is obvious. The authors claim, after extended investigation, to have produced an active vitamine product from corn, autolysed yeast and orange juice, thereby assuring the presence of the antineuritic, antirachitu, and antiscorbutic factors.

Composition.

Unfortunately, the exact method of preparation is not available, but it is stated that the product is stable, concentrated, and can be readily added to any ordinary or temporary diets. An analysis is given showing the constituents to be calcium oxide 10 per cent., phosphorus 15 per cent., nitrogen 3.5 per cent., fat 2.5 per cent., iron 0.3 per cent., silicates 5.6 per cent., and water 10 per cent. The bulk is made up of a double compound of magnesium and calcium with inosite of phosphoric acid, but such details convey but little useful knowledge until further facts are published.

The point, however, lies in the fact that this product can be prepared, kept, and added at will to the diets of unfavourably placed children. The first experiments included pigeons, in which it was found that one grain of the vitamine compound, given every other day, in addition to a polished rice diet, was sufficient to prevent (*a*) the onset of polyneuritis, (*b*) to cure polyneuritis, and (*c*) to maintain and increase the body weight.

Later the tests were applied to children. Cases were chosen showing typical evidence of malnutrition, rickets, and marasmus, and the babies nursed in separate cubicles under the constant supervision of day and night nurses. All food taken was carefully weighed or measured and the children's own weight was systematically recorded at short intervals. A series of nitrogen, phosphorus, and calcium estimations were run to appreciate the exact changes in metabolism which occurred, and as a control a set of babies were nursed under exactly similar supervision but taking exactly the same diet as they had when first coming under observation. Definitely good results are claimed, in that in the first group of four children, in whom vitamine was mixed with their food, all showed such marked improvement after feeding with the added vitamine product that they could be considered practically normal.

In a second group children received daily doses of vitamine, separate apparently from their ordinary food, and, although not behaving so well as those of the first group, they rapidly gained in weight, and completely surpassed those to whom no additional vitamine had been given.

In any case, the experiments give yet another proof, if that were needed, of the paramount importance of the accessory food factors, but it has also, if the authors' claims to absolute stability are justified, an important bearing upon the possibility of efficiently remedying vitamine deficiency when the so-called "fresh" food products, etc., are not available. In that sense, therefore, attention is drawn to their work and comments and the importance of a product which contains in lasting concentrated form all *three* of the active accessory food factors.

